# The importance of optical coherence tomographic sectioning in a case with unilateral subtle visual disturbance related to a vertical intraretinal hyperreflective line

**DOI:** 10.22336/rjo.2025.88

**Published:** 2025

**Authors:** Özlem Özkan, Ziya Ayhan, Ali Osman Saatci

**Affiliations:** 1 Department of Ophthalmology, Dokuz Eylül University, Izmir, Turkey

**Keywords:** intraretinal hyperreflective line, macula, microperimetry, optical coherence tomography, retinal disease, BCVA = Best-corrected visual acuity, IHL = Intraretinal hyperreflective line, MacTel-2 = Macular telangiectasia type 2, OCT = Optical coherence tomography, OCTA = Optical coherence tomography angiography, SD-OCT = Spectral-domain OCT, SS-OCT = Swept-source OCT

## Abstract

A 60-year-old otherwise healthy man presented with the complaint of missing letters while reading with his right eye. Best-corrected visual acuity (BCVA) was 10/10 in both eyes. Slit-lamp examination was unremarkable, and the intraocular pressure was within normal range bilaterally. Multimodal imaging was performed, and spectral domain and swept source optical coherence tomography (OCT) revealed a foveolar vertically oriented intraretinal hyperreflective line (IHL) in the right eye. Microperimetric examination revealed a lower fixation stability and macular integrity in the right eye compared to the left. The patient recalled an episode of a severe blow to his head a few years before. The intraretinal hyperreflective line is an optical coherence tomographic finding that is often detectable only with careful tomographic sectioning. The present case underscores the importance of careful OCT sectioning and multimodal imaging to elucidate the cause of even subtle visual disturbances.

## Introduction

Intraretinal hyperreflective line (IHL) is an optical coherence tomography finding that is most often detected by careful foveolar volume analysis, and its presence likely indicates a reaction to photoreceptor, Muller cell, and/or retinal pigment epithelial damage [**1,2**].

Although IHLs mainly were described in eyes with vitreomacular surface disorders and during the healing process following the macular hole surgeries [**3-8**] they were also noted in various retinal pathologies including acquired vitelliform lesions, pattern dystrophies, fundus flavimaculatus, MacTel-2, commotio retina, exudative age related macular degeneration, posterior uveitic entities, Coats’ disease, diabetic macular edema and central retinal arterial occlusion [**1,2,9-13**].

We present the detailed multimodal imaging features of a case with 10/10 bilateral vision and unilateral reading difficulties due to a vertical right foveolar IHL without apparent cause.

## Case presentation

A 60-year-old otherwise healthy man presented with the complaint of missing letters while reading with his right eye. On examination, best-corrected Snellen visual acuity (BCVA) was 10/10 in both eyes. Slit-lamp examination was unremarkable, and the intraocular pressure was within normal range bilaterally.

**Fig. 1:**
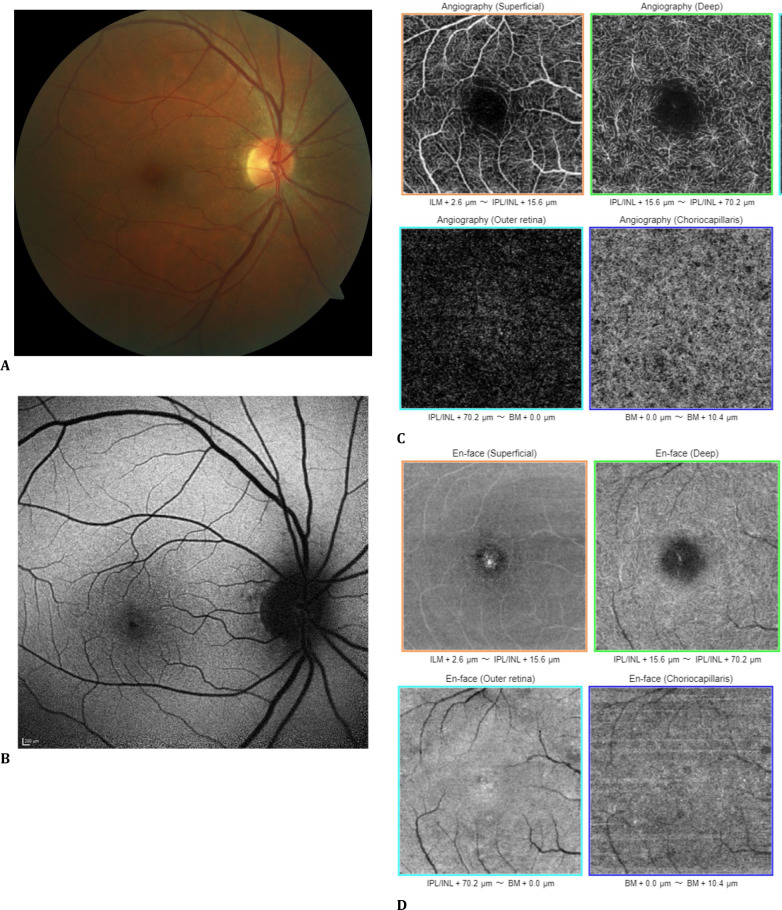
Right eye (**A**) Color fundus photograph depicting some peripapillary retinal pigment epithelial changes; (**B**) Almost unremarkable fundus autofluorescence image; (**C**) 6 x 6 mm optical coherence tomography angiographic (OCTA) scans’ angiography and (**D**) en-face OCT sections exhibiting normal flow pattern

**Fig. 2:**
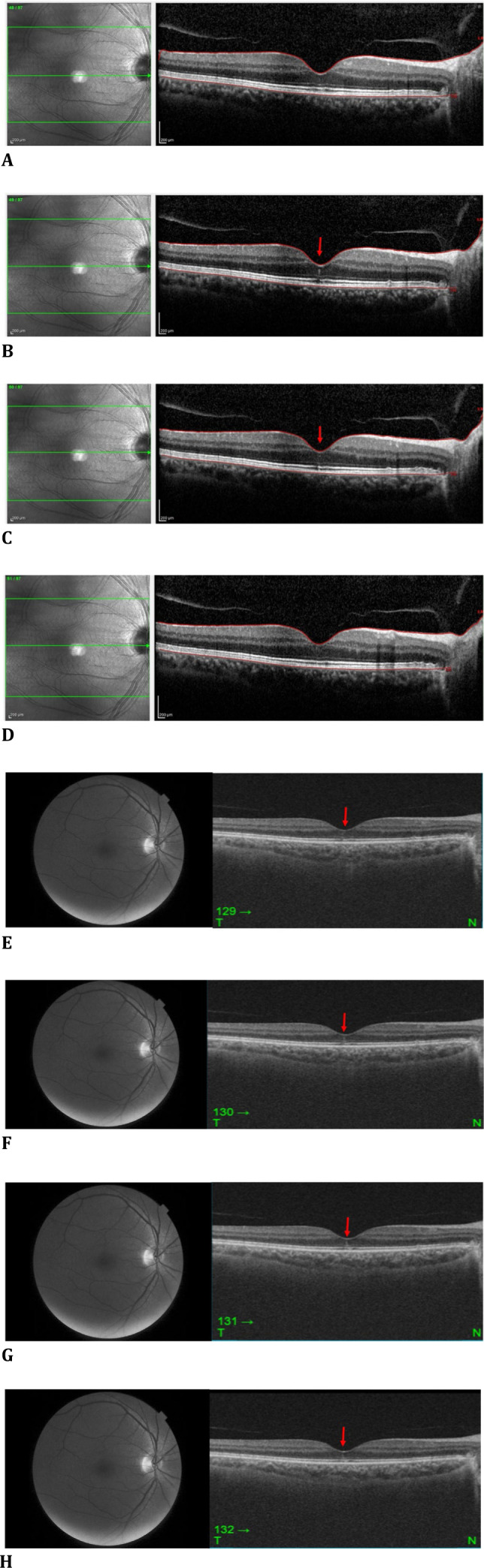
Right eye (**A-D**) SD-OCT sections with a 61 μm spacing between the consecutive scans depicting the presence of vertical IHL only in two sections (arrows); (**E-H**) SS-OCT sections with a 27 μm spacing between the successive scans depicting the presence of vertical IHL only in four sections (arrows)

Multimodal posterior segment imaging was performed. The left eye appeared normal on every imaging modality. There were a few peripapillary pigmentary changes in the right eye (**Fig. 1A**), and the autofluorescence image was almost unremarkable (**Fig. 1B**). 6 x 6 mm optical coherence tomography angiography (OCTA) and en-face OCT sections exhibited normal flow in the right fovea (**Fig. 1C, D**). There was a vertical hyperreflective line extending from the ellipsoid zone (EZ) to the outer nuclear layer (ONL) in the central foveola, detected by both spectral-domain OCT (SD-OCT; Spectralis®, Heidelberg Engineering, Heidelberg 2, Germany) with a distance between consecutive scans of 61 μm (**Fig. 2A-D**) and by swept-source OCT (SS-OCT, DRI OCT Triton Plus®; Topcon Corporation, Tokyo, Japan) (**Fig. 2E-H**), with a distance between consecutive scans of 27 μm. There was also evidence of vitreomacular adhesion on OCT scans.

**Fig. 3:**
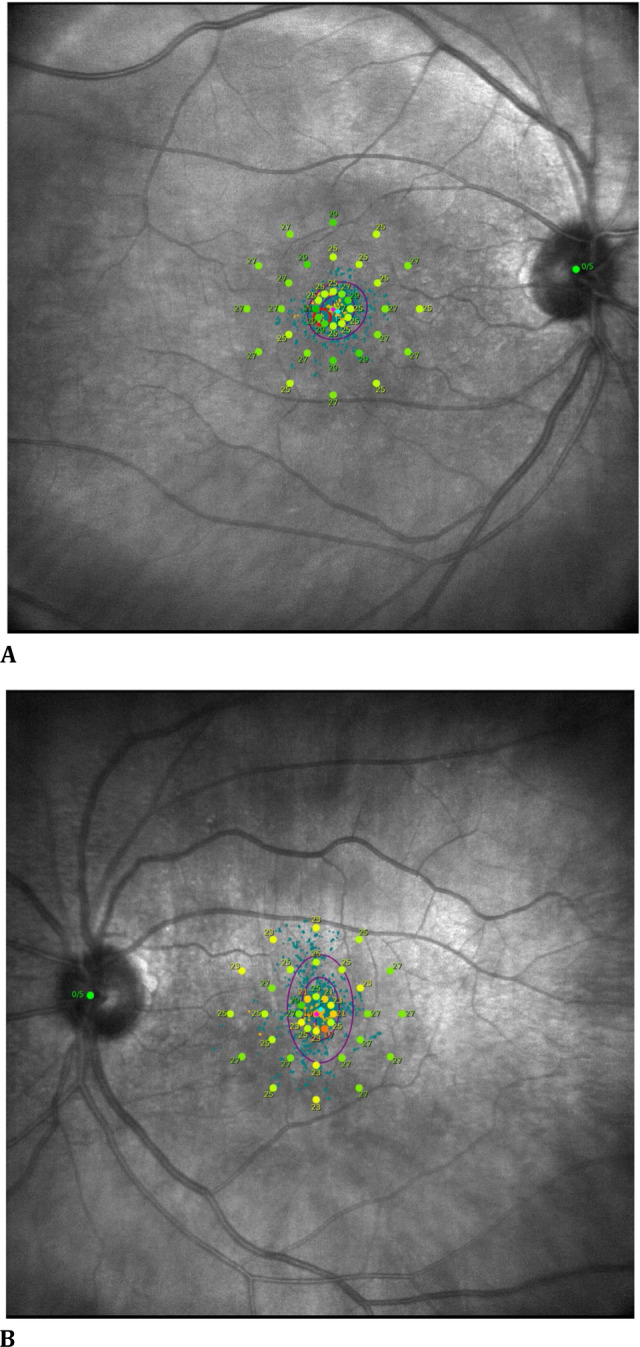
Microperimetry examination depicting a more crowded and slightly shifted central fixation point (**A**) in the right eye compared to (**B**) the left eye

Microperimetry examination revealed a lower fixation stability in the right eye (P1:71%, P2:85%), whereas P1:78% and P2:94% were in the left eye. While macular integrity was 80.2% in the right eye, it was 95.8% in the left eye. The central fixation point in the right eye was more crowded and slightly displaced when compared to the left eye (**Fig. 3A, B**).

The patient was reexamined three months later, and both the visual acuity and multimodal imaging features were unchanged.

## Discussion

IHL was defined as a vertical line extending from the ellipsoid zone band through the outer nuclear layer to the internal limiting membrane in the foveola. However, the clinical and visual implications of its presence are still unknown. The present case exhibited an IHL in the right eye, with a complaint of difficulty reading despite near visual acuity. The most likely cause was presumably a past blunt trauma, as the patient recalled a severe blow to his head that occurred a few years before.

Blunt trauma may result in outer retinal layer damage due to shock waves traversing the eye from the anterior impact site. Li et al. [**14**] reviewed the medical records of 21 patients with contusion maculopathy and found a vertical IHL in only one eye, with a good visual outcome. Amoroso and colleagues [**1**] reported the features of IHL in 49 eyes from 43 patients, collected from three tertiary eye centers. The most common etiology was adult vitelliform dystrophy or pattern dystrophy (24 of 49 eyes). The IHLs fully disappeared in cases of hemorrhage, multiple evanescent white dot syndrome, or after resolution of vitreomacular traction, but usually persisted with gradual thinning in other conditions. Kayabasi et al. [**2**] reported 40 eyes of 38 patients with IHL in a cross-sectional study. In only a single eye, the cause was commotio retinae. However, no information was available regarding the patient’s visual status.

The spacing between OCT sections is crucial. The distance between two consecutive scans was 61 microns on SD OCT and 27 microns on SS OCT in our case, and IHL could be detected on only two successive SD OCT sections, whereas on four consecutive SS OCT sections. The present case underscores the importance of careful OCT sectioning and multimodal imaging to elucidate the cause of even subtle visual disturbances.

## Conclusion

The OCT sign of foveolar intraretinal hyperreflective line can even occur after a subtle blunt trauma, and its presence can only be documented by multimodal imaging techniques, especially by careful OCT sectioning.
